# Role of MIF/CD74 signaling pathway in the development of pleural mesothelioma

**DOI:** 10.18632/oncotarget.7314

**Published:** 2016-02-11

**Authors:** Cintia D'Amato-Brito, Davide Cipriano, Didier J. Colin, Stéphane Germain, Yann Seimbille, John H. Robert, Frédéric Triponez, Véronique Serre-Beinier

**Affiliations:** ^1^ Department of Thoracic and Endocrine Surgery, University Hospitals and University of Geneva, Geneva, Switzerland; ^2^ MicroPET/SPECT/CT Imaging Laboratory, Centre for BioMedical Imaging (CIBM), University Hospitals and University of Geneva, Geneva, Switzerland; ^3^ Cyclotron Unit, University Hospitals and University of Geneva, Geneva, Switzerland

**Keywords:** cancer, pleura, mesothelioma, macrophage migration inhibitory factor (MIF), CD74

## Abstract

Macrophage migration inhibitory factor (MIF) is a pro-inflammatory cytokine implicated in acute and chronic inflammatory diseases. MIF is overexpressed in various tumors. It displays a number of functions that provide a direct link between the process of inflammation and tumor growth. Our group recently identified the MIF-receptor CD74 as an independent prognostic factor for overall survival in patients with malignant pleural mesothelioma.

In the present study, we compared the levels of expression of MIF and CD74 in different human mesothelioma cell lines and investigated their physiopathological functions *in vitro* and *in vivo*.

Human mesothelioma cells expressed more CD74 and secreted less MIF than non tumoral MeT5A cells, suggesting a higher sensitivity to MIF. In mesothelioma cells, high MIF levels were associated with a high multiplication rate of cells. *In vitro*, reduction of MIF or CD74 levels in both mesothelioma cell lines showed that the MIF/CD74 signaling pathway promoted tumor cell proliferation and protected MPM cells from apoptosis. Finally, mesothelioma cell lines expressing high CD74 levels had a low tumorigenic potential after xenogeneic implantation in athymic nude mice.

All these data highlight the complexity of the MIF/CD74 signaling pathway in the development of mesothelioma.

## INTRODUCTION

Malignant pleural mesothelioma (MPM) is an aggressive cancer of the inner lining of the chest cavity which develops mainly after inhaling asbestos fibers. According to the WHO classification, malignant mesothelioma is either classified as epithelioid (mostly composed of epithelial-shaped cells), sarcomatoid (mostly composed of spindle-shaped cells), or biphasic (composed of both types of cells) [1]. In 2007, the WHO estimated that about 125 million people around the world were exposed to asbestos at work, and that at least 90,000 people died each year from asbestos-related diseases. In the next decades, MPM incidence will continue to increase, even in regions where the commercial use of asbestos has been banned (Europe, Australia and Japan) and will contribute to cancer mortality in countries lacking working protection and/or persisting with its use (Asia and India). While surgery is a valid option for patients with early stage MPM, most patients with locally advanced invasive disease are not amenable to surgical resection [2] and treatment is palliative chemotherapy combining cisplatin and pemetrexed. While this treatment may relieved symptoms, it provides only modest survivals, since median survival averages only 9–18 months from the time of diagnosis.

The exact mechanisms involved in the neoplastic transformation of mesothelial cells are still unknown. Identifying key genes related to the underlying oncogenic processes and understanding the malignant mesothelial cell regulation pathways are essential to develop more effective treatments. Initial tumor growth depends on increased cell proliferation and reduced cell death, both of which are stimulated by inflammation-driven mechanisms. Tumor cells trigger an intrinsic inflammatory response that builds up a protumorigenic microenvironment [3]. No study has precisely characterized the tumor inflammatory environment in the early development and growth of mesothelioma. Macrophage migration inhibitory factor (MIF) is a pro-inflammatory cytokine involved in both innate and adaptive immunity. Originally described as a T cell-derived product, MIF is released by numerous cell types and is involved in many inflammatory and autoimmune diseases [4]. Furthermore, MIF may be involved in cell proliferation and differentiation and several studies have reported increased MIF mRNA levels in tumor cells and pre-tumor states in prostate [5], colon [6–8], and hepatocellular cancers [9], adenocarcinomas of the lung [10], glioblastomas [11, 12] and melanomas [13]. Several groups have shown a correlation between MIF expression and cancer prognosis in hepatocellular carcinomas, colon and prostate cancers [8, 14, 15]. The invariant chain or CD74 was the first MIF surface receptor described [3]. Chemokine receptors CXCR2 and CXCR4 have also been shown to be MIF receptors [16–18]. In murine models of human colorectal adenoma [16] and metastatic breast cancer [19], inhibition of MIF expression by genetic deletion or RNA interference decreased tumor progression and metastasis. A reduction of tumor growth was also observed in sub-cutaneous human neuroblastoma [20] and prostate cancer xenografts [21], after inhibition of MIF expression by MIF antisens transfection or RNA interference or after treatment with anti-MIF antibodies or MIF inhibitors. Such a decrease of MIF activity was associated with inhibition of tumor angiogenesis [21].

Our group has recently shown that the majority of malignant mesothelial tumor cells express MIF and its receptor CD74, with a homogenous distribution between the different histological subtypes [22]. We also demonstrated that high levels of CD74 were an independent prognostic factor for prolonged overall survival in MPM patients. To clarify the potential involvement of MIF in MPM, we investigated the expression and the role of MIF and its receptors (CD74, CXCR2 and CXCR4) in viability, proliferation and *in vivo* tumorigenesis of human MPM cells.

## RESULTS

**Figure 1:**
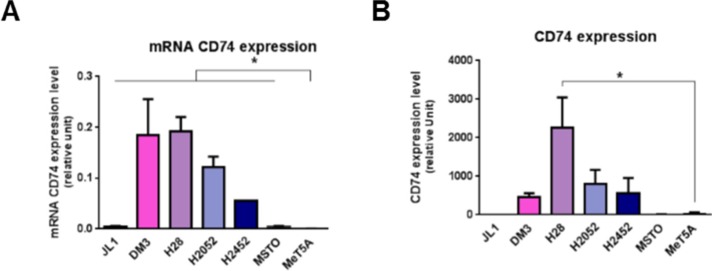
Mesothelioma cell lines overexpress CD74 MIF-receptors CD74 mRNA (**A**), and total protein (**B**) were measured in human immortalized normal mesothelial cell line (MeT5A) and different mesothelioma cell lines (JL-1, DM-3, H28, H2052, H2452, MSTO). Relative mRNA (A) or protein (B) expression levels were measured by qPCR and western blotting respectively. Data represent the mean values (± SD) of 3 to 6 independent experiments. significant difference Kruskal-Wallis test between normal mesothelial cell line MeT5A and mesothelioma cell lines: *P < 0.05.

**Figure 2:**
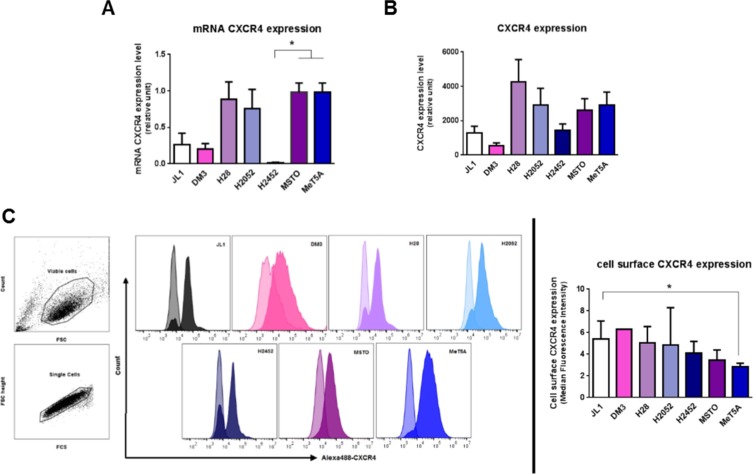
Mesothelioma cell lines express CXCR4 MIF-receptors CXCR4 mRNA (**A**), total protein (**B**) and cell surface protein (**C**) were measured in human immortalized normal mesothelial cell line (MeT5A) and different mesothelioma cell lines (JL-1, DM-3, H28, H2052, H2452, MSTO). Relative mRNA (A) or protein (B) expression levels were measured by qPCR and western blotting respectively. Data represent the mean values (± SD) of 3 to 6 independent experiments. CXCR4 distribution on the cell surface was analysed by flow cytometry (C). Cells were treated with EDTA and stained with anti-CXCR4 antibody followed by Alexa488-conjugated anti-IgG. Controls received equivalent concentrations of isotype-matched IgG. Viable mesothelial and mesothelioma cells were first gated according to SSC-A vs FSC-A scatted plot and doublet were excluded using a pulse geometry gate FSC-H × FSC-A plot (C, left panel). For all histograms, data are shown as cell number vs. the relative fluorescence. The light-coloured histogram depicts isotype control, whereas the dark-coloured one represents CXCR4 antibody. Each histogram shows data from a single representative experiment although each analysis was repeated at least seven times. CXCR4 expression was normalized according to the median fluorescence intensity with the isotype-matched IgG (C, right panels). Kruskal-Wallis test between normal mesothelial cell line MeT5A and mesothelioma cell lines: *P < 0.05.

**Figure 3:**
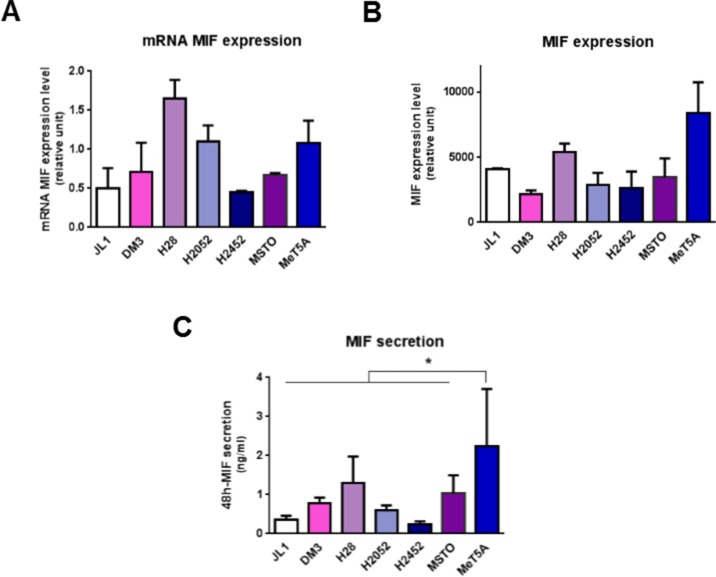
Mesothelioma cell lines express MIF MIF mRNA (**A**) total protein (**B**) and secretion levels (**C**) were measured in human immortalized normal mesothelial cell line (MeT5A) and different mesothelioma cell lines (JL-1, DM-3, H28, H2052, H2452, MSTO). Relative mRNA (A) or total protein (B) expression levels were measured by qPCR and western blotting respectively. MIF concentrations in 48 h-cultured media were measured by ELISA (C). Data represent the mean values (± SD) of 3 to 6 independent experiments. Kruskal-Wallis test between normal mesothelial cell line MeT5A and mesothelioma cell lines: *P < 0.05.

In order to identify the role of MIF in MPM, mRNA and protein levels of MIF-receptors CD74 (Figure 1), CXCR2 and CXCR4 (Figure 2) and MIF (Figure 3), were assessed in six different human MPM cell lines of different histological types (JL-1, DM-3, H28, H2052, H2452 and MSTO) and in an non tumorigenic immortalised mesothelial cell line MeT5A (Table 1).

**Table 1:** Mesothelioma cell lines studied

Cell line	JL-1	DM-3	H28	H2052	H2452	MSTO
Histological type of original tumor	Epithelioid	Sarcomatoid	Not done	Not done	Biphasic	Biphasic
Reference	[42]	[42]			[45]	[45]

### MPM cell lines expressed higher levels of CD74 compared to mesothelial cell line MeT5A

Expression of CD74 in MeT5A cells was weak to absent (Figure 1A and 1B). CD74 mRNA expression levels of all MPM cell lines studied were significantly higher than that of MeT5A cells (Figure 1A, *n* = 3; *P* < 0.05). CD74 total protein expression in MPM cell lines was higher than that of MeT5A cells except for JL-1 and MSTO cell lines (Figure 1B). Cell surface expression of CD74 was not detected using flow cytometry in all MPM cell lines studied and MeT5A cells (Supplementary Figure S1). Previous studies about cell surface CD74 showed that surface expression of newly synthesized CD74 complexes concern only few percents of cellular CD74 and is followed by a rapid internalization to the endosomal pathway [27] complicating cell surface detection of these complexes.

Thus, CD74 appeared to be primarily expressed in malignant mesothelial cells, indicating that such tumor cells may be prone to stimulation with MIF.

### MPM cell lines expressed similar levels of CXCR4 than mesothelial cell line MeT5A

CXCR4 mRNA and protein levels (Figure 2A and 2B) were assessed by RT-qPCR and western blotting. No significant difference in CXCR4 expression levels between MPM cell lines compared to MeT5A was observed. A difference in cell surface expression of CXCR4 (*P* < 0.05) was detected between MeT5A and the MPM JL1 cells (Figure 3C) with a median fluorescence intensity of 2.9 ± 0.3 (*n* = 7) and 5.4 ± 1.7 (*n* = 8) for MeT5A and JL1 respectively. In MPM cell lines, CXCR4 expression levels varied between the different cell lines studied and these levels of expression were not related to the histological type of the MPM.

The chemokine receptor CXCR2 has also been described as a receptor for MIF. CXCR2 mRNA levels were weak to absent in the MeT5A cells as well as MPM cells (data not shown) suggesting a very poor protein expression of CXCR2 in MPM and mesothelial cells.

### MPM cell lines secreted lower levels of MIF compared to mesothelial cell line MeT5A

MIF mRNA and protein levels (Figure 3A and 3B) were assessed by RT-qPCR and western blotting. No significant difference in MIF expression levels between MPM cell lines compared to MeT5A was observed. In MPM cell lines, MIF expression levels varied between the different cell lines studied and these levels of expression were not related to the histological type of the MPM.

During tumorigenesis, tumor cells secrete growth factors and cytokines (including MIF) which amplify tumor cell transformation, activate their proliferation and modify the activation state of surrounding inflammatory cells. In order to determine whether MeT5A and MPM cells spontaneously secrete MIF in the culture medium, secreted MIF levels by MeT5A and MPM cell lines were measured after 48 h-culture by ELISA (Figure 3C). In all MPM cell lines studied, accumulated MIF concentrations in the supernatant reached levels significantly lower (from 2-fold to 14-fold lower for H28 and JL-1 respectively, *n* = 5; *P* < 0.05) than the MIF secretion rate of MeT5A.

In summary, most MPM cell lines expressed higher levels of CD74 and secreted lower levels of MIF than mesothelial cell line MeT5A suggesting a higher sensitivity to MIF.

### MPM cells expressing high levels of MIF/CD74 and secreting high levels of MIF showed high multiplication rate

We compared physiological characteristics, such as cell multiplication, cell proliferation and apoptosis of MeT5A cells with JL-1 and H28 MPM cell lines chosen in regard to their MIF/CD74 expressions and MIF secreting levels. As shown previously, JL-1 cells expressed the lowest MIF/CD74 levels and secreted the lowest MIF level and H28 cells expressed the highest MIF/CD74 levels and secreted the highest MIF level (Figures 1 and 3) of the six MPM cell lines studied.

**Figure 4:**
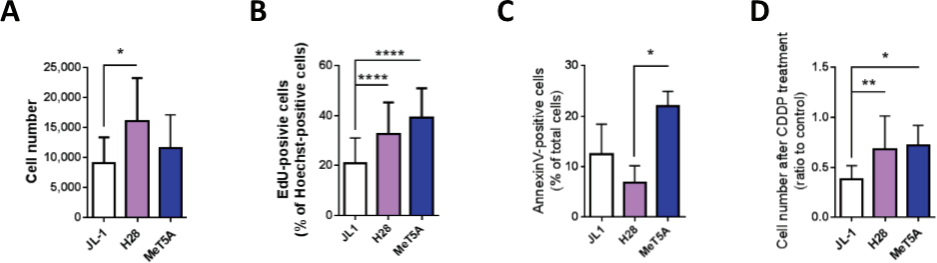
High levels of MIF and CD74 in mesothelioma cells are associated with high multiplication rate JL-1, H28 and MeT5A cells were cultured for 24 h. Cell multiplication (**A**) was measured using a MTT assay. Cell proliferation (**B**) was estimated by EdU incorporation after 2 h cultured with EdU. Proliferation rate was calculated as the percentage of EdU^+^-Hoechst^+^ double-positive cells (Zeiss Apotome, 20× magnification, Axiovision 4.6). Cell apoptosis (**C**) was analyzed using Annexin-V-PE/7-AAD double staining. Effect of cisplatin on cell multiplication (**D**) was assessed using the MTT assay performed on mesothelioma cells cultured for 24 h with 100 mmol/L of cisplatin. Control was cells cultured for 24 h without cisplatin. Bars are mean values (± SD) for *n* = 4–15 independent experiments. Kruskal-Wallis test between normal mesothelial cell line MeT5A and mesothelioma cell lines: *P < 0.05, **P < 0.01, ***P < 0.001 and ****P < 0.0001.

The multiplication rate of the three cell lines was evaluated using a mitochondrial activity assay (MTT). Two days after plating 5 × 10^3^ cells per well, cell number of MeT5A cells was not different than the number of JL-1 and H28 mesothelioma cells. H28 cell number was two-fold higher than the number of JL-1 cells (Figure 4A; 11, 595 ± 5, 532 cells, *n* = 9, for MeT5A; 9, 058 ± 4, 336 cells, *n* = 9, for JL-1 and 16, 136 ± 7, 098 cells, *n* = 15, for H28, *P* < 0.05) indicating a higher multiplication rate for H28 cells. The population doubling time of MeT5A (18.9 ± 4.5 h (*n* = 4)) was the lowest one compared to that of H28 (29.1 ± 2.7 h (*n* = 7; *P* < 0.01)) and JL-1 (41.0 ± 9.1 h (*n* = 6; *P* < 0.01)). The population doubling time of H28 cells was statistically lower than that of JL-1 cells (*P* < 0.05). It is well described that cancerous cell populations exhibit abnormally high cell proliferation and enhanced apoptotic cell death [23–25]. As the rate of tumor growth depends in part on an excess of proliferation over apoptosis, we determined and compared proliferation and apoptosis rates of JL-1, H28 and MeT5A cell lines. Statistically significant increasing proliferation rates (Figure 4B) were observed between JL-1 (20.7 ± 10.3% of EdU-positive cells, *n* = 62, *P* < 0.0001), H28 (32.6 ± 13.0% of EdU-positive cells, *n* = 73) and MeT5A cells (39.3 ± 11.6% of EdU-positive cells, *n* = 48).

The level of apoptosis of H28 cells was lower than the level of apoptosis of MeT5A cells (Figure 4C and Table 2; 6.8 ± 3.4% of total H28 cells *vs* 21.9 ± 3.0% of total MeT5A cells; *n* = 4; *P* < 0.05). The level of apoptosis of JL-1 cells tended to be lower than of MeT5A cells and slightly higher than that of H28 cells (Figure 4C; 12.5 ± 5.9% of total JL-1 cells, *n* = 4). Necrosis, evaluated as the percentage of 7AAD-positive cells, was similar between JL-1, H28 and MeT5A cell lines (5.0 ± 5.3%, 1.4 ± 0.5% and 2.1± 2.6%, respectively) (data not shown).

**Table 2:** Cell apoptosis of MeT5A, JL-1 and H28 cell lines expressed in % of total cells

	MeT5A			JL-1				H28			
	Mean	SD	*n*	Mean	SD	*n*	*p vs* MeT5A	Mean	SD	*n*	*p vs* MeT5A
Cell apoptosis (AnnexinV-positive cells)	21.9	3.0	4	12.5	5.9	4	ns	6.8	3.4	4	< 0.05
Early apoptosis (AnnexinV-pos and 7AAD-negative cells)	14.2	2.5	4	7.6	4.1	4	ns	4.3	2.5	4	< 0.05
Late apoptosis death (AnnexinV-pos and 7AAD-positive cells)	7.8	2.3	4	5.0	2.1	4	ns	2.5	1.0	4	< 0.05

Finally, chemosensitivity of the three cell lines to cisplatin, a chemotherapeutic drug used in human MPM treatment was assessed. Cell multiplication was evaluated after 24 h of treatment with cisplatin using a MTT assay (Figure 4D). H28 and MeT5A cells were less sensitive to cisplatin treatment than JL-1 cells as after 24 h of cisplatin treatment, 67.9 ± 33.4% (*n* = 12) of H28 and 71.8 ± 20.1% (*n* = 9) of MeT5A cells and only 38.1 ± 13.5% (*n* = 9) of JL-1 cells recovered compared to untreated cells.

All these data were analysed in regard to MIF-secreted level and CD74 expression in JL-1, H28 and MeT5A cell lines (Figures 1 and 3). Human MPM cell line H28 (expressing high level of CD74 and secreting high level of MIF) showed a high level of multiplication and proliferation rate associated with a low level of apoptosis rate. In contrast, the MPM cell line JL-1 (expressing low level of CD74 and secreting low level of MIF) showed a low level of multiplication and proliferation rate associated with a high level of apoptosis rate. These data suggested that MIF increased cell viability through increase of proliferation and decrease of apoptosis of MPM cells. Additionally, high level of secreted MIF and CD74 expression was also associated to a lower sensitivity to cisplatin.

### MIF or CD74 deficiency impacts H28 and H2052 cell proliferation and apoptosis

**Figure 5:**
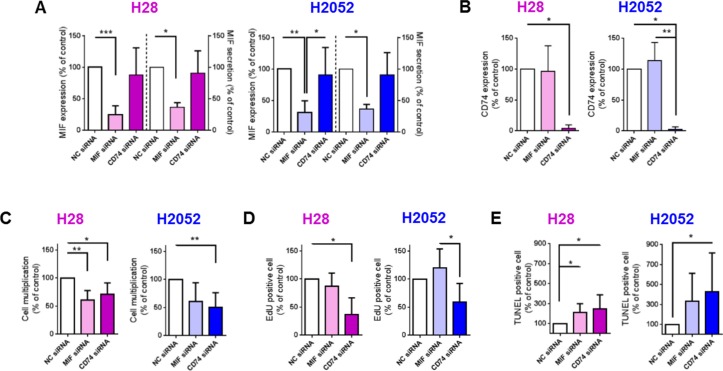
MIF and CD74 promote H28 and H2052 cell multiplication *in vitro*, increasing cell proliferation and decreasing cell apoptosis H28 (pink bars) and H2052 (blue bars) cells transfected with NC-siRNA, MIF-siRNA and CD74-siRNA were assessed after 48 h of culture, for MIF expression (**A** left panel), MIF secretion (A right panel) and CD74 expression (**B**) by western blot and ELISA. The multiplication of transfected H28 and H2052 cells (**C**) was evaluated by total cell counting (Hoechst-positive nuclei) after 48 h of culture. Proliferation (**D**) and apoptosis (**E**) rates of transfected H28 and H2052 cells were evaluated by EdU incorporation and TUNEL assay, respectively. Data represent the mean values (± SD) for *n* = 4–8 independent experiments. Kruskal-Wallis test between cells transfected with NC, MIF or CD74 siRNAs: *P < 0.05, **P < 0.01, and ***P < 0.001.

In order to assess whether MIF binding to CD74 explained differences in multiplication (proliferation and apoptosis) of MPM cells, we investigated cell multiplication, proliferation and apoptosis of H28 and H2052 cells after small interfering RNA (siRNA) down-regulation of MIF or CD74. H28 and H2052 cells transfected with a non-coding (NC) siRNA were used as control cells. siRNA down-regulation of MIF led to a reduction of MIF protein expression of 75% and 68% for H28 and H2052 cells respectively (Figure 5A, left panel; MIF expression in MIF siRNA treated cells was 25 ± 14% and 32 ± 18% of MIF expression in NC siRNA treated cells in H28 and H2052 cells respectively, *n* = 7 to 8; *P* < 0.001 and *P* < 0.01) and a reduction of MIF secretion of 41% and 63% in H28 and H2052 respectively (Figure 5A, right panel; MIF secretion by MIF siRNA treated cells was 59 ± 13% and 37 ± 7% of MIF secretion by NC siRNA treated cells for H28 and H2052 respectively; *n* = 5 to 7, *P* < 0.05). The same results were obtained by transfecting MPM cells with two different siRNA specific for MIF (Hs_MIF_5 or Hs_MIF_6) individually or by pooling them. Multiplication rate of MIF siRNA treated-cells was reduced of 39% and 40% for H28 and H2052 respectively, compared to that of NC siRNA treated cells (Figure 5C, 61 ± 17% and 60 ± 34% for H28 and H2052 respectively, *n* = 6–7, *P* < 0.01). This reduction in cell multiplication was explained by a reduction of 13% of cell proliferation of MIF siRNA-treated H28 cells (Figure 5D, 87 ± 24%, *n* = 5 for H28 cells; 120 ± 34%, *n* = 6 for H2052 cells) and an increase of 213% and 334% of cell apoptosis for H28 and H2052 (Figure 5E, 213 ± 86% and 334 ± 278% for H28 and H2052, *n* = 7–13, *P* < 0.05).

CD74 expression of H28 and H2052 cells treated with CD74 siRNA was reduced by 96% and 98%, respectively (Figure 5B; 4 ± 6%, *n* = 4 for H28, and 2 ± 4%, *n* = 6 for H2052; *P* < 0.05). The same results were obtained transfecting H28 and H2052 cells with two different siRNA specific for CD74 (Hs_CD74_2 or Hs_CD74_5) individually or by pooling them. Multiplication rate of H28 and H2052 transfected with CD74 siRNA was reduced of 29% and 49% respectively, compared to that of NC siRNA treated cells (Figure 5C, 71% ± 20%, *n* = 6, *P* < 0.05 for H28; 51 ± 26%, *n* = 7; *P* < 0.01 for H2052). CD74 siRNA-treated H28 and H2052 cells showed a decrease in cell proliferation compared to NC siRNA treated cells (Figure 5D; 37 ± 29%, *n* = 5, *P* < 0.05 for H28; and 59 ± 33%, *n* = 6 for H2052) and an increase in cell apoptosis (Figure 5E; 245 ± 143%, *n* = 4 for H28 and 427 ± 389%, *n* = 7 for H2052; *P* < 0.05).

In summary, in human MPM H28 and H2052 cell lines, a MIF expression and secretion reduction led to a decrease in cell multiplication due to an increase of cell apoptosis. Additionally, a reduction in CD74 expression led to a decrease of cell multiplication, due to a decrease of cell proliferation and an increase of cell apoptosis.

### MPM cells expressing higher level of CD74 showed lower tumorigenic potential

In order to analyse the association between MIF/CD74 stimulated pathway and the tumorigenic potential of MPM cells, we followed xenografts development of two human MPM cell lines expressing high levels of CD74 (H28 and H2052, see Figure 1B), and two human MPM cell lines expressing low levels of CD74 (JL-1 and MSTO, see Figure 1B). Cell lines were injected individually sub-cutaneously (s.c.) and intra-pleuraly (i.pl.) into athymic nude mice (immunodeficiency affecting T cells), and tumor development were assessed using caliper (for s.c. tumors) and positron emission tomography/computed tomography (PET/CT) scans (for pleural tumors).

**Figure 6:**
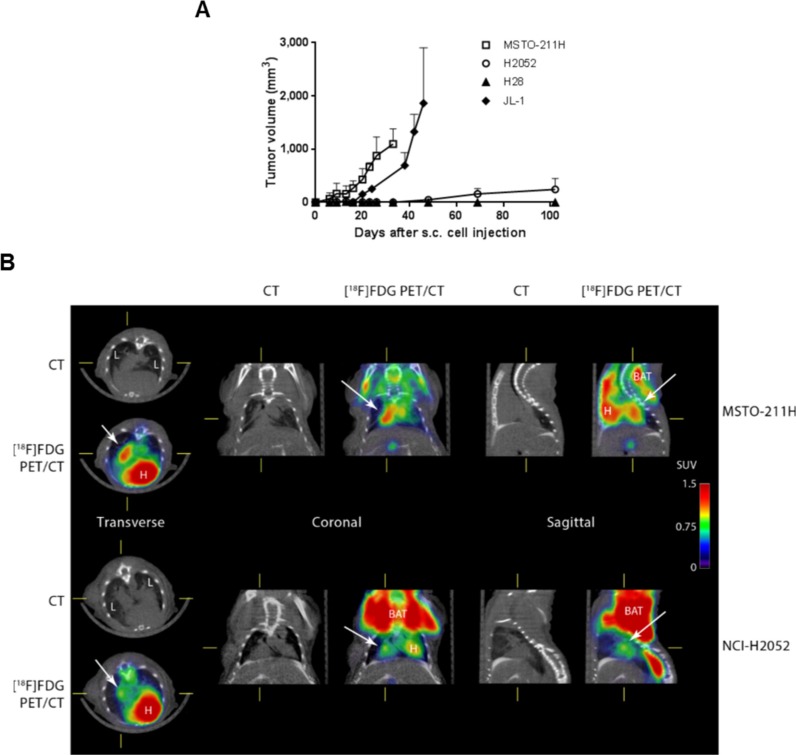
The tumorigenic potential of mesothelioma is negatively associated to CD74 levels Human mesothelioma cells (MSTO, JL-1, H2052 and H28) were injected s.c. (1 × 10^6^ cells) and i.pl. (1 × 10^6^ cells) into athymic nude mice (*n* = 4–6 per group). Sub-cutaneous tumor growth rates were assessed using caliper measurements (**A**). Intra-pleural tumors were followed using [^18^F] FDG-PET/CT. Representative PET/CT of MSTO (at 23 days post i.pl. injection) and H2052 tumors (at 69 days post i.pl. injections) in nude mice are shown (**B**). The images shown were trans-axial slices containing the FDG-avid tumors and organs, with CT (gray scale) providing anatomic references and PET (pseudo-color scale) showing the location and intensity of high tumor and organ glucose utilization. CT: CT mediastinal window; [^18^F]FDG PET/CT: PET–CT fused image; White arrows indicated mesothelioma tumors. L = lung, H = heart, BAT = brown adipose tissue.

MSTO, JL-1 and H2052 cell lines formed s.c. tumors (Figure 6A) in nearly all injected mice (10/10; 7/7 and 5/6, respectively). MSTO and JL-1 cell lines generated fast-growing s.c. tumors in 100% injected mice (Figure 6A). MSTO injected mice were euthanized 33 days-post injection at the first clinical signs of distress. Sub-cutaneously JL-1 tumors were explanted 46 days-post injection. At these time points, the mean volume for s.c. tumors was 1,097 ± 286 mm^3^ (*n* = 6) for MSTO and 1,863 ± 1,042 mm^3^ (*n* = 5) for JL-1 tumors. Growth of s.c. H2052 xenograft was slower than that of MSTO and JL-1 xenografts. The first identified H2052 tumors were measured at day 48-post injection (49 ± 56 mm^3^, *n* = 6). At day 102-post injection, H2052 injected mice showed clinical signs of distress and were euthanized. The mean volume for s.c. H2052 tumors at the end of the experiment was 244 ± 205 mm^3^ (*n* = 4). No mice injected s.c. with H28 cells developed tumors until 155 days after cell injection.

**Figure 7:**
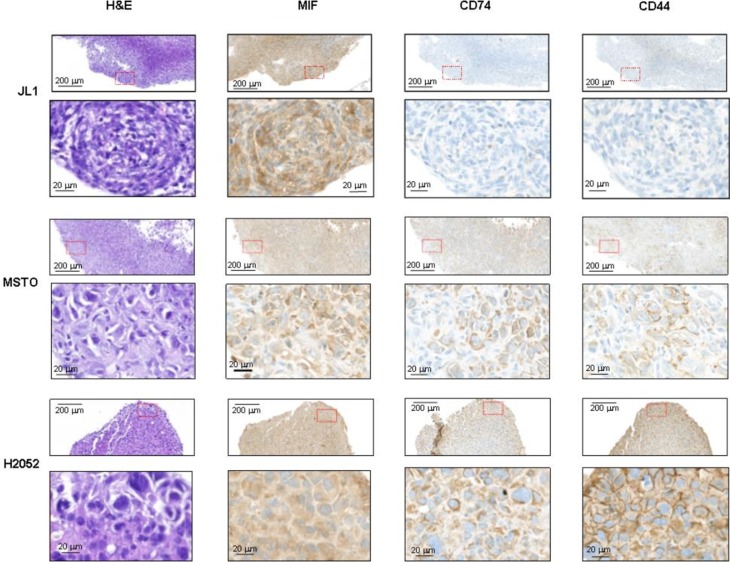
MIF, CD74 and CD44 expressions in intra-pleural human mesothelioma Human mesothelioma cells (JL-1, MSTO, and H2052) were injected i.pl. (1.10^6^ cells) into athymic nude mice (*n* = 4–6 per group). Representative photomicrographs of JL-1 (**A**), MSTO (**B**) and H2052 (**C**) intra-thoracic tumors stained with H & E, anti-MIF, anti-CD74 and anti-CD44 antibody. Representative photomicrographs are shown (red box magnified in the bottom panel). Scale bars: 200 μm and 20 μm.

MSTO, JL-1 and H2052 pleural tumors were identified in the thoracic cavity using 2-deoxy-2-[^18^F] fluoro-D-glucose ([^18^F]FDG)-PET/CT analyses (Figure 6B, top panels) in nearly all injected mice (9/10, 5/10 and 5/6, respectively). Post-mortem examination showed that athymic nude mice injected with MSTO, JL-1 and H2052 cells had extensive pleural tumors. Most of tumors were free in the thoracic cavity; some tumors were attached to the thoracic muscles and the diaphragm and few to the lung. No mice injected i.pl. with H28 cells developed tumors until 155 days after injection. In order to estimate the MIF and CD74 expression in intra-pleural MPM tumors developed into athymic nude mice, we performed immunohistochemical staining for MIF, CD74 and the co-receptor of CD74, CD44 on intra-pleural JL-1, H2052 and MSTO tumors. As shown in Figure 7, cytoplasmic MIF was detected in tumor cells of JL-1, H2052 and MSTO tumors. As expected neither CD74 nor its co-receptor CD44 were detected in JL-1 tumor cells. Cytoplasmic and membranous expression of CD74 was detected in H2052 tumor cells. These cells also expressed high amount of cytoplasmic and membranous CD44. Cytoplasmic and membranous CD74 was also detected in MSTO tumor cells as well as CD44. CD44 labeling of MSTO tumors was less intense than that of H2052.

Our *in vivo* data suggested that CD74/CD44 expression was negatively associated with *in vivo* tumorigenesis in MPM.

## DISCUSSION

MIF is a pro-inflammatory cytokine overexpressed in various cancers [5–10, 12, 13]. It may play an important role in carcinogenesis by promoting cell proliferation, tumor angiogenesis and metastasis [26]. Up to now, no study has addressed the role of MIF in MPM. Recently, we have shown that the majority of the malignant mesothelial tumor cells expressed MIF and its receptor CD74, with a homogenous distribution between the different histotypes. We also demonstrated that high expression of CD74 was an independent prognostic factor for prolonged overall survival in MPM patients [22].

In this study, we characterized the expression and impact of MIF on human MPM cell lines. MIF protein expression was confirmed in immortalized mesothelial MeT5A cell line and in several MPM cell lines derived from epithelioid, sarcomatoid and biphasic human MPM. MIF protein expression level was not significantly different between MeT5A and MPM cells, suggesting that *de novo* synthesis and intracellular protein storage did not differ between normal and malignant mesothelial cells. In contrast, MIF secretion was lower in MPM cells compared to MeT5A cells. These lower levels of secreted MIF could be related to the higher level of expression of MIF-receptor CD74 in MPM cells, conferring a higher sensitivity to MIF of MPM cells compared to normal mesothelial cells. The very low level of CD74 in normal mesothelial cell line MeT5A suggest that these cells are not sensitive to MIF, explaining in part why their high MIF secretion level was not correlated to their multiplication rate. Cell surface CD74 could not be detected on MPM cells using flow cytometry. Previous studies about cell surface CD74 showed that surface expression of newly synthesized CD74 complexes concern only few percents of cellular CD74 and is followed by a rapid internalization to the endosomal pathway [27] complicating cell surface detection of these complexes. Expression of the chemokine receptors CXCR2 and CXCR4, described as MIF receptors, was characterized as the same levels in mesothelial and MPM cell lines suggesting that these receptors are not implicated in MPM cell multiplication.

In the MPM cell lines studied, we observed that the higher CD74 expression and MIF secreted levels, the higher the multiplication rate was, suggesting that MIF signaling through CD74 could play a role in MPM cell proliferation and apoptosis. This role was confirmed by reducing MIF and CD74 expression in MPM H28 and H2052 cells using specific siRNA. A reduction in CD74 expression led to a decrease in the multiplication rate due to a decrease of the proliferation and an increase of the apoptosis rates. Reducing MIF expression and secretion had the same effect on multiplication suggesting that MIF signal transduction is initiated by binding to CD74. MIF reduction did not change the proliferation rate. This could potentially be explained either by a resting MIF level sufficient to activate proliferation of MPM cells or by pro-stimulatory effect of another CD74 ligand such as the functional homolog of MIF, the D-dopachrome tautomerase [28].

Our data suggest that secreted MIF (or MIF homolog) from tumor cells has pro-stimulatory and anti-apoptotic effects on MPM cells. This pro-stimulatory effect could also be due, *in situ*, to MIF secreted by cells from the tumor microenvironment, such as tumor-associated macrophages, tumor-infiltrating lymphocytes, or cancer-associated fibroblasts [29–33]. In several tumoral cells (breast, colon, prostate, lung, …), MIF signaling in cancer is well described to be triggered by its receptor CD74 [21, 34, 35]. Previously, in tumoral cells of human MPM explants, we showed [22] a co-expression of MIF and CD74 suggesting that MIF effect on MPM cells was triggered in part by binding to CD74. In summary, in MPM cell lines, activated MIF/CD74 pathway has a pro-tumorigenic function by increasing tumor cell proliferation and protecting them from apoptosis.

*In vivo* data obtained from the development of MPM into athymic nude mice showed at contrary that the lower CD74 expression level is, the higher the tumorigenic potential is. These results should be related to our previous results obtained on human tissue array [22] showing that low CD74 expression level in MPM cells was associated with a low patient survival rate. We also observed an up-regulation of CD74 expression *in situ* in MSTO-211H tumors. These data therefore showed additional regulatory pathway on CD74 expression *in vivo*. These different results highlight the complex role of MIF and its receptor CD74 in tumor growth. The effect of MIF and CD74 on carcinogenesis seems to change with the cell type as well as the stage of the cancer. This may be a consequence of the activation of different signaling pathways. Several studies reported the activation of the extracellular signal regulated kinase (ERK)1 and 2 in the mitogen-activated protein kinase pathway, and the PI3K/Akt/SRC signal transduction cascade [3, 36, 37] subsequently to the binding of MIF to its receptor complex CD74/CD44. These activated pathways upregulate cell proliferation, decrease cell apoptosis and enhance cell migration [21, 38]. In contrast, other studies showed that MIF can activate the AMPK pathway, leading in some cancers to a decrease in cell proliferation, cell viability and in their metastatic potential [39, 40]. Finally, we observed differences in tumor development after s.c. or i.pl. injection with H2052 cells. H2052 tumors developed slowly but extensively in the thoracic cavity while s.c tumors poorly grew. These differences could be due to differences in the vascularization, growth factors and immune cells in the environment of these both sites. While s.c graft site has the advantage to be easily accessible, it is not representative of the environment in which the tumor originated. Wang Y *et al.* [41] showed that the histology of human prostate tumors implanted into immunodefficient mice was best in the orthotopically grafted samples and that s.c. grafted tissues had the poorest profile of histopathologic differentiation. The orthotopic graft site provides a tumor microenvironment that closely reflects the clinical situation. The use of orthotopic sites versus other sites may well be important in the metastatic spread patterns of any advance malignant graft. The use of the orthotopic thoracic site for xenografting has not been widespread, due largely to the technical difficulties in reaching, and monitoring tumor development in this location. In our study, the progression of mesothelioma in the pleural cavity was easily monitored by using PET/CT imaging, offering a new valuable tool for evaluation of anticancer therapy for MPM. In the future, in order to clarify the complex role of MIF and/or CD74 from tumor and stromal cells in MPM development, we plan to perform i.pl. syngeneic implantations of murine mesothelioma cells deleted or not in MIF or CD74 expression into wild-type or MIF-deficient or CD74-deficient mice and study tumor development, tumor angiogenesis and identify the inflammatory cells recruited by the host.

In conclusion, the results of the present study showed that most human MPM cell lines expressed higher level of CD74 and secreted lower level of MIF than MeT5A mesothelial cell line. Decreasing MIF or CD74 expression in H28 and H2052 MPM cells reduced multiplication rate of the tumor cells due to a reduction in proliferation and an increase in apoptosis. Finally, *in vivo* data following xenogeneic human MPM cells implanted into athymic mice suggested that CD74 expression was negatively associated with *in vivo* tumorigenesis in MPM. All these data showed the complex role of MIF/CD74 pathway on MPM development with, on one hand, a promoting effect on tumor cell viability and on the other hand, a promoting effect on mesothelioma cell-stroma interactions.

## MATERIALS AND METHODS

### Cell culture

The non-tumorigenic immortalized mesothelial cell line MeT5A and the MPM cell lines H28 (NCI-H-28), H2052 (NCI-H2052), MSTO (MSTO211H) and H2452 (NCI-H2452) were purchased from American Type Culture Collection. The MPM cell lines JL-1 and DM-3 were established and characterized in our laboratory from human biopsies [42]. Cells were routinely cultured in RPMI 1640 medium containing 10% (v/v) fetal calf serum (complete RPMI, *Life Technologies*). Cultures were grown at 37°C in 5% CO_2_.

### RNA interference

H28 and H2052 cells were transfected twice. A reverse transfection was first performed followed by a forward transfection 48 h latter, according to the manufacturer's instructions with 67 nmol/l of commercially available MIF (Hs_MIF_5 FlexiTube siRNA ref. SI02781065; Hs_MIF_6 FlexiTube siRNA ref. SI02781247), CD74 (Hs_CD74_2 FlexiTube siRNA ref. SI00063049; Hs_CD74_5 FlexiTube siRNA ref. SI03058405), or nonspecific scramble siRNA oligonucleotides (AllStars Negative Control siRNA ref. 1027281) from Qiagen using INTERFERin(^®^) transfection reagent (Polyplus). Downregulation of MIF and CD74 expressions was measured 48 h after transfection by RT-qPCR and immunoblot analysis. MIF secretion, cell viability and proliferation and cell apoptosis were measured 48 h after transfection.

### Total RNA isolation and real-time RT-PCR

The expression of *MIF*, *CD74*, *CXCR2*, *CXCR4, GAPDH, GUSB*, *EEFLA1* and *TBP* mRNAs was evaluated by quantitative RT-PCR analysis. Total mRNAs from each cell lines was extracted by Qiagen RNEasy Midi extraction Kit (Qiagen) according to manufacturer's instructions.

Reverse transcription was performed from 0.5 μg of total RNA using PrimerScript reverse transcriptase enzyme (Takara bio inc. Kit) and a mix of random hexamers – oligo d(T) primers, following suppliers instructions.

Real-time RT-PCR was performed on each sample in triplicate performed from 500 ng cDNA using an ABI 7900HT SDS system with Power SYBR Green PCR Master Mix (Applied Biosystems, Foster City, California). SYBR green primers were designed using the program Primer Express v 2.0 (Applied Biosystems) with default parameters and obtained from Invitrogen. Primer sequences for the targeted human genes are available upon request. Results were normalized to the expression levels of *GAPDH, GUSB*, *EEFLA1* and *TBP* expression genes, used as housekeeping genes. Normalisation factor and fold changes were calculated using the GeNorm method [43].

### Cell lysis and western blotting analysis

Cell lysates for western blotting were prepared in sample buffer 1× (62.5 mmol/l Tris-HCl pH 7.4, 2% (w/v) SDS, 10% (v/v) glycerol, and 1% (v/v) β-mercaptoethanol) supplemented with protease inhibitor cocktail (Roche Molecular Diagnostics), and 10 mmol/l phosphatase inhibitors sodium orthovanadate (Sigma) and sodium pyrophosphate (Sigma). Protein concentrations of all samples were determined with the amido black method [44] or using a colorimetric assay (BCA Protein Assay Kit, Pierce). Total protein extracts (5 to 25 μg were loaded on a SDS-PAGE gel. Electrophoresed samples were electroblotted onto polyvinylidene fluoride (PVDF) membranes (Immobilon-P, Millipore) in the presence of 0.01% (w/v) SDS and 20% (v/v) methanol. The membranes were saturated for 1 h at room temperature (RT) in a 10 mmol/l Tris-HCl buffer (pH 7.4) containing 150 mmol/l NaCl, 0.1% (v/v) Tween-20, and 5% (w/v) milk (TBS/T/milk), and then incubated overnight at 4°C with rabbit polyclonal primary antibodies diluted in TBS/T/milk at 1/1000 for GAPDH (#2118, Cell Signaling Technology), 1/200 for CXCR4 (ab2074, Abcam), 1/1000 for CD74 (HPA010592, Sigma) and 1/1000 for MIF (BR47, from the Roger lab, Lausanne, Switzerland). Detection was performed using an anti-rabbit HRP-conjugated secondary antibody (Bio-Rad Laboratories) and an enhanced chemiluminescence detection system (ECL Plus western blotting detection reagents from Amersham, GE Healthcare). Quantifications were then performed using a ChemiDoc XRS and Quantity One software (Bio-Rad Laboratories).

### Flow cytometry

Cell populations were analyzed with a BD Accuri C6 instrument (Becton-Dickinson). Data were analyzed with FlowJo (Tree Star). Adherent cells were detached using EDTA 0.5 mM, washed and resuspended in staining buffer (PBS, FBS 3%, EDTA 5 mM). Cells were stained with PE-conjugated anti-CD74 antibody (LN2, Biolegend) for 30 min at 4°C. CXCR4 was stained with rabbit anti-human CXCR4 antibody (ab2074, Abcam) for 30 min at 4°C followed by Alexa488-conjugated anti-rabbit IgG (A11008, Invitrogen) for 30 min. at 4°C. Controls received equivalent concentrations of isotype-matched IgG.

### Immunohistochemistry analysis

4 μm thick mesothelioma tumor sections from formalin fixed paraffin embedded samples were analysed by immunohistochemistry using anti-MIF (gift of Thierry Roger, Lausanne), anti-CD74 (HPA010592, Sigma) and anti-CD44 antibodies (HPA005785, Sigma) using the Ventana Discovery automated staining system (Ventana Medical Systems, Tucson, AZ, USA). Ventana reagents for the entire procedure were used. Antigenicity was retrieved by heating slides in CC1 cell conditioning solution for 20 min (EDTA antigen retrieval solution pH 8.4; 20 min for CD74 and CD44, 36 min for MIF). After automatic deparaffinization and heating, slides were incubated 30 min at 37°C with primary antibodies diluted at 1/300 (MIF), 1/1000 (CD74) and 1/500 (CD44) in antibody diluent from Dako (S2022). Detection of anti-MIF, anti-CD74 and anti-CD44 antibodies were performed using the rabbit OmniMap kit (760–149).

### Biochemical analyses

Thirty thousand cells were seeded onto 24-well microplates in 500 μl/well of complete RPMI medium. Supernatants were removed 48 h after culture, centrifuged 20 min at 1,000 g and stored at −20°C. MIF levels in medium supernatant were detected using the Human MIF Duoset kit (R & D System).

### Cell viability

Cell viability was determined by the reduction of 3-(4, 5-dimethylthiazol-2-yl)-2, 5-diphenyltetrazolium bromide (MTT, Sigma-Aldrich). Five thousand cells were seeded onto 96-well microplates in complete RPMI. After overnight incubation, cells were serum-starved for 24 h in RPMI medium supplemented with 1% (w/v) human serum albumin (HSA). Then, medium was replaced and cells were cultured in complete RPMI with or without 100 mmol/l of cisplatin Ebewe^®^ (Sandoz Pharmaceuticals SA) for 48 h at 37°C. MTT solution (500 μg/ml in RPMI/1% HSA) was added for 2 h at 37°C. Absorbance was read in a spectrophotometer at 570 nm.

### Cell proliferation

Cell proliferation was measured by performing 5-ethynyl-20-deoxyuridine (EdU) incorporation assay, using the Click-iT EdU imaging kit (Life Technologies) according to the manufacturer's instructions. Cells were cultured in 8-chamber slides (Lab-Tek permanox chamber slide from Nunc) at a density of 25 × 10^3^ cells/cm². After overnight incubation, and serum-starvation for 24 h in RPMI medium supplemented with 1% (w/v) HSA, medium was replaced by complete RPMI, and cells were incubated for another 24 h at 37°C. Then, 5 μM of EdU was added to each chamber and cells were cultured for additional 2 h at 37°C. The cells were fixed with 10% (v/v) formalin for 20 min (RT) and permeabilized with 0.5% (v/v) Triton X-100 for 10 min (RT). After washing with PBS, cells were incubated with the EdU reagent cocktail for 30 min at RT. Then, the cells were stained with mounting medium *ProTaqs Mount Fluor (BIOCYC*) containing Hoechst33342. The EdU (green) and Hoechst (blue) positive nuclei were counted and the EdU incorporation rate was expressed as the ratio of EdU positive cells to total Hoechst positive cells.

### Cell apoptosis analysis

Cell apoptosis was evaluated using Annexin-V-phycoerythrin/7-amino-actinomycin D (Annexin-V-PE/7-AAD) double staining. Cells (2 × 10^5^ to 4 × 10^5^) were cultured for 24 h in non-adherent 35 mm-dishes, in complete RPMI. After washes with cold PBS and Annexin V Binding Buffer (100 mmol/l Hepes, 1.4 mol/l NaCl, 25 mmol/l CaCl_2_), cells were resuspended in 100 μl of Annexin V Binding Buffer and incubated with 2.5 μl of PE Annexin V (BioLegend #640912) and 7 μl of 7-AAD (BD Pharmingen # 51-2359KC) for 15 min at RT in the dark. Annexin V Binding Buffer (100 μl) were added to each tube and annexin and 7-AAD fluorescences were analysed with an Accuri C6 flow cytometer (BD). For each measurement, 5 × 10^4^ cells were counted. Dot plots and histograms were analysed by BD Accuri^™^ C6 software (BD). Annexin V-positive cells were considered in the early stage of apoptosis; Annexin V- and 7-AAD-positive cells were considered in the late stage of apoptosis or necrotic. Annexin V- and 7-AAD-negative cells correspond to the viable cell fraction.

H28 cell apoptosis after transfection with siRNA was evaluated using a terminal deoxynucleotidyl transferase dUTP nick-end labeling (TUNEL) assay. Forty-eight hours after transfection, H28 cells were fixed in 10% (v/v) formalin and TUNEL assay (*In Situ* Cell Death Detection Kit, TMR red, Roche Diagnostics) was performed according to the manufacturer's instructions. Briefly, cells were permeabilized in 0.1% (v/v) triton-X100 in PBS for 10 min and incubated with the reaction mixture containing the enzyme terminal deoxynucleotidyl transferase and the fluorescent TMR-conjugated dUTP for 1 h at 37°C. Labeled DNA was visualized by fluorescence microscopy.

### Sub-cutaneous and i.pl. implantations of MPM cells in nude mice

Mice were anaesthetized with isoflurane and MPM cells (MSTO, JL-1, H2052 and H28) were injected s.c. (1 × 10^6^ tumor cells suspended in 200 μl of of a matrix containing 30% of RPMI and 70% of HyStem-C hydrogel (ESI BIO, Alameda, CA, USA)) on the right dorsa and into the left pleural cavity (1 × 10^6^ tumor cells suspended in 50 μl of RPMI) of 8-week-old athymic female nude mice nu/nu (Harlan) (*n* = 5 or 6 per group). Once a week, volumes of s.c. tumors were measured using calipers and calculated using the following formula:

(Long axis (mm) × short axis (mm) × short axis (mm)/2

Intra pleural tumors were followed by 2-deoxy-2-[^18^F]fluoro-D-glucose ([^18^F]FDG)-PET/computed tomography (CT) scans. PET/CT was performed using a Triumph PET/SPECT/CT system (Trifoil, Chatsworth, CA, USA). The mice were fasted for 12 h and blood glucose was measured before each scan. Mice were anesthetized with 2% isoflurane and were i.v. injected retro-orbitally with 5–6 MBq of [^18^F]FDG. Mice were then left awake at RT during an uptake time of 60 min. 10 min prior to PET scan, mice were injected intraperitoneally with 700 μL of 132 mg/ml meglumine ioxitalamate (Telebrix, 6% m/v iodide, Guerbet AG, Zürich, Switzerland) to delineate the abdominal region and subjected to CT scans. Images were obtained at 80 kVp, 160 μA, and 1024 projections were acquired during the 360° rotation with a field of view of 71.3 mm (1.7 × magnification). After 60 min of [^18^F]FDG uptake, PET scans were started for a duration of 20 min. PET scans were reconstructed with the built-in LabPET software using an OSEM3D (20 iterations) algorithm and images were calibrated in Bq/ml by scanning a phantom cylinder. The Triumph XO software, which uses a back-projection engine, was used to reconstruct the CT scans with a matrix of 512 and a voxel size of 0.135 mm. CT scans were co-registered with the PET scans using the plugin Vivid (Trifoil) for Amira (FEI, Hillsboro, OR, USA) and exported as dicom files. The software Osirix (Pixmeo, Bernex, Switzerland) was used to quantitatively analyse the datasets and generate pictures.

At the end of the experiment, all mice were euthanized and closely examined for the presence of thoracic tumors. This study was conducted under protocols revised and approved by the institutional animal care and use committee and by Geneva's veterinarian state office.

### Statistics

Results were presented as means ± SD. Statistical differences among three or more groups were examined by a Kruskal-Wallis test. Differences between pairs of groups were examined for statistical significance using the unpaired Mann-Whitney *U* test. A *P* value < 0.05 was considered as statistically significant.

## SUPPLEMENTARY MATERIALS FIGURE


