# Supplementary material for: Role of MIF/CD74 signaling pathway in the development of pleural mesothelioma
Source: Oncotarget. 2016 Feb 11;7(10):11512–25. doi: 10.18632/oncotarget.7314 (PMC4905490; doi:10.18632/oncotarget.7314)
Supplement: Supplementary file 1 [file oncotarget-07-11512-s001.pdf]

Role of MIF/CD74 signaling pathway in the development of pleural mesothelioma

Supplementary Materials

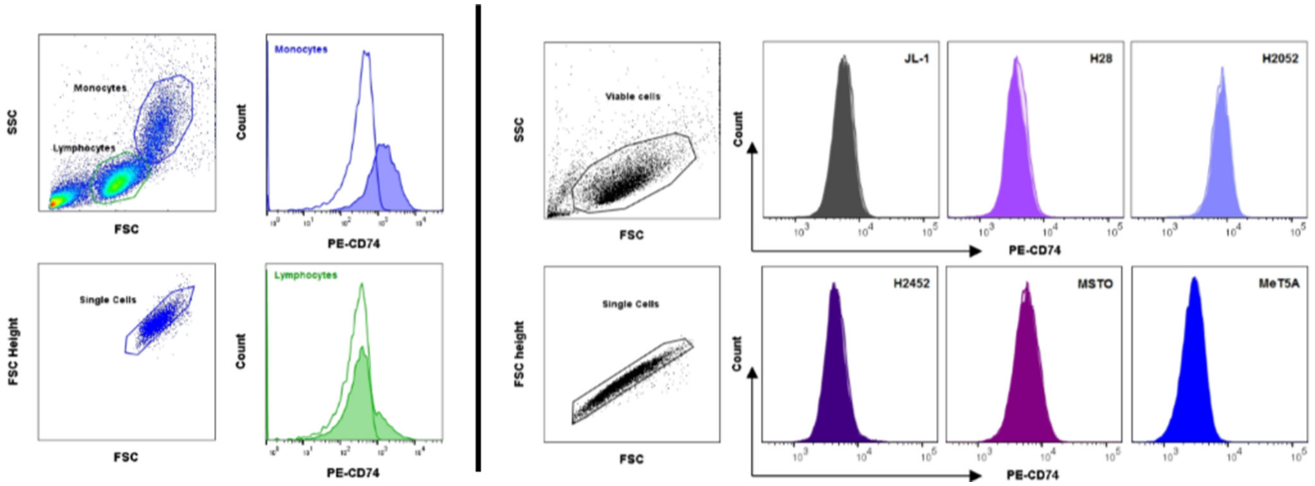

Supplementary Figure S1: Cell surface CD74 distribution on MPM cells. The cell surface distribution of CD74 was analysed by flow cytometry. Cells were treated with EDTA and stained with PE-conjugated anti-CD74 antibody. Controls received equivalent concentrations of isotype-matched IgG. CD74 cell surface expression on human monocytes and lymphocytes was shown as positive control (left panel). Monocytes, lymphocytes and viable mesothelioma cells were first gated according to SSC-A vs FSC-A scatted plot and doublet were excluded using a pulse geometry gate FSC-H x FSC-A plot. For all histograms, data are shown as cell number vs. the relative fluorescence. The clear histogram depicts isotype control, whereas the full one represents PE-CD74 antibody. Each histogram shows data from a single representative experiment although each analysis was repeated at least three times.
